# Electrons for intraoperative radiotherapy in selected breast-cancer patients: late results of the Montpellier phase II trial

**DOI:** 10.1186/1748-717X-8-191

**Published:** 2013-08-01

**Authors:** Claire Lemanski, David Azria, Sophie Gourgou-Bourgade, Norbert Ailleres, Aurelie Pastant, Philippe Rouanet, Pascal Fenoglietto, Jean-Bernard Dubois, Marian Gutowski

**Affiliations:** 1 Department of Radiation Oncology and Medical Physics, I.C.M. – Institut regional du Cancer Montpellier, INSERM U896, Montpellier cedex 5, F-34298, France; 2 Unit of Biostatistics, I.C.M. - Institut regional du Cancer Montpellier, Montpellier, France; 3 Department of Oncology and Reconstructive Surgery, I.C.M. - Institut regional du Cancer Montpellier, Montpellier, France

## Abstract

**Background:**

The Montpellier cancer institute phase II trial started in 2004 and evaluated the feasibility of intraoperative radiotherapy (IORT) technique given as a sole radiation treatment for patients with an excellent prognostic and very low recurrence risk.

**Methods:**

Forty-two patients were included between 2004 and 2007. Inclusion criteria were patients ≥ 65 years old, T0-T1, N0, ductal invasive unifocal carcinoma, free-margin > 2 mm. IORT was delivered using dedicated linear accelerator. One fraction of 21 Gy was prescribed and specified at the 90% isodose using electrons. *In vivo* dosimetry was performed for all patients. Primary end-point was the quality index. Secondary endpoints were quality of life, local recurrences, cosmetic results, specific and overall survival.

**Results:**

At inclusion, median age was 72 years (range, 66–80). Median tumor diameter was 10 mm. All patients received the total prescribed dose. No acute grade 3 toxicities were observed. Late cosmetic results were good at 5 years despite the poor agreement of accuracy assessment between patients and physicians. Four patients (9.5%) experienced a local failure and underwent salvage mastectomy. The 5 year-disease free survival is 92.7% (range 79.1−97.6). All patients are still alive with a median follow-up of 72 months (range 66–74).

**Conclusion:**

Our results confirm with a long-term follow-up that exclusive partial breast IORT is feasible for early-breast cancer in selected patients. IORT provides good late cosmetics results and should be considered as a safe and very comfortable “one-step” treatment procedure. Nevertheless, patient assessments are essential for long-term quality results.

## Background

Breast-conserving surgery followed by a whole-breast postoperative RT (WBRT) is widely considered to be the current standard of care for patients with early breast cancer. This treatment leads to an excellent local tumor control with local recurrence rates around 6% after 10 years, particularly in the very-low risk subgroup [1]. This classical 5–7 weeks WBRT is simple and safe in the large majority of patients but may bring some local early and late side effects. In addition, it is frequently inconvenient for women, namely in the elderly, who sometimes prefer to omit it assuming the risk of local recurrence. During the last decades, through numerous observational series and large clinical trials, more than 80% of the local relapses occurred in the same quadrant in this clinical situation [2].

On this basis, reducing both the treatment volume and duration was considered to be an innovative approach and was developed under the name of accelerated partial breast irradiation (APBI). Intraoperative radiotherapy (IORT) is one of them that offers the advantages of an excellent delineation of the tumor bed under visual control, a very good dose homogeneity, and a high normal tissue sparing [3,4].

Alongside our IORT experience delivered as a boost [5], we started in 2004 the RADELEC phase II trial evaluating IORT as a sole treatment in very low-risk patients. We report here the long-term results of safety, cosmesis, and carcinologic events.

## Patients and methods

This study was conducted in accordance with the declaration of Helsinki and the local institutional review board. All patients provided written informed consent.

### Inclusion criteria

Between November 2004 and November 2007, 96 women accepted to participate in the RADELEC phase II trial evaluating IORT delivered to the tumor bed as exclusive radiotherapy. Inclusion criteria were T1N0M0 [6] unifocal ductal invasive carcinoma (biopsy-proven) with positive (> 10%) estrogen receptors, non-metastatic disease, and age ≥ 65 years old. No extensive intraductal (EIC) or lymph vessel invasion had to be identified on primary biopsy.

Local evaluation (mammography and breast ultrasound) evaluated precisely the diameter of the tumor (MRI was optional). Lobular tumors were excluded due to the risk of multifocality. Neoadjuvant treatments were not allowed before surgery.

### IORT procedure and postoperative therapies

The detailed procedure was described in a previous article [7]. Briefly, IORT modalities included a dedicated linear accelerator (Saturne 43, Varian, France) located centrally between the six operating theaters. Lumpectomy was performed with an incision centered on the tumor or periareolar in outer or inner/medial lesions, respectively. Tumor-free margins of at least 2 mm were assessed by frozen sections. Axillary lymph node dissection was performed using a sentinel lymph node procedure in all cases. Nodes were analyzed by intraoperative imprint cytology. The tissue surrounding the excision cavity was then mobilized and approximated by sutures to bring it into the radiotherapy planning target volume. The radiation oncologist identified visually the tumor bed and, together with the surgeon, measured the depth of the tissue to be irradiated. A semi-conductor detector (PTW) was placed in the middle of the tumor cavity and fixed to proceed to an *in vivo* dosimetry. The applicator tubes were then placed under visual control. The entire tumor bed was strictly encompassed customarily with a 20-mm margin using 40–60 mm diameter flat-ended brass applicators. Complete skin sparing was verified in each case.

The treatment was delivered at using electrons with an energy ranged between 6 and 9 MeV, in accordance with the thickness of breast tissue to be treated. All patients received 21 Gy, prescribed and specified at the depth of the 90% isodose line, which was defined as the optimal and maximal tolerated dose level [8].

*In vivo* dosimetry was performed in all patients. Following IORT, the incision was closed in the conventional fashion by the surgeon.

Patients were seen three weeks after IORT for the first medical follow-up and were prescribed adjuvant treatment according to our guidelines. Postmenopausal women who were shown to have estrogen receptor and/or progesterone receptor-positive tumors (ER and/or PR = 10% of the tumor cells positive by an immunocytochemical assay) were prescribed an aromatase inhibitor in front line or sequentially after two years of tamoxifen. No adjuvant chemotherapy was initially planned in this setting i.e. T1N0M0 and age ≥ 65 years old.

### Long-term cosmetic assessment

Long term cosmetic results were yearly performed by the surgeons and the radiation oncologists with clinical exams, systematic photographs (two pictures with frontal and profile views), and a local questionnaire filled in independently by the patient and the physician. We considered the breast shape and position, the areola shape, and the presence or absence of telangiectasia.

### Statistical analysis

This phase II trial was conducted using a two-stage optimum Simon design with forty-two eligible patients required for evaluation of the primary endpoint defined as IORT reproducibility using the quality index (QI) comparing prescribed and the *in vivo* measured doses [7].

Data were summarized by frequency and percentage for categorical variables, and by means, standard deviations, median, and range for continuous variables. Updated median follow-up was obtained with the reverse Kaplan-Meier method.

All survival events were measured from the day of surgery to event (assessed by clinical exam, mammography, or breast ultrasound). Disease-free survival (DFS; event was locoregional/distant recurrence or death) and overall survival (OS; event was death) rates were estimated according to the Kaplan-Meier method.

Cohen's kappa coefficient was used to measure inter-rater agreement for qualitative items [9]. A κ less than 0 is a disagreement, from 0.0 to 0.20 is a very poor agreement, from 0.21 to 0.40 is a poor agreement, from 0.41 to 0.60 is a moderate agreement, from 0.61 to 0.80 is a strong agreement, and from 0.81 to 1.00 is an almost perfect agreement.

Analyses were performed using STATA 11.0.

## Results

### IORT reproducibility as a primary end-point

Among the 42 patients, 36 procedures were assessable and 35 were measured as acceptable according to the primary endpoint (97%). The median measured dose was 23 Gy (range, 19.6–26.5 Gy), with a high concordance with the prescribed dose (21 Gy at the 90% isodose, corresponding to 23 Gy at the 100% level).

### Long-term toxicities and carcinologic events

Among the 94 patients who accepted to participate in this trial and signed the informed consent before the surgical procedure, 11 patients were treated by IORT but were secondary excluded of the final analysis due to the definitive pathologic report. The reasons of non IORT delivery were detailed for 41 patients in Lemanski et al. [7]. Finally, 42 patients were included in the RADELEC trial according to the study criteria.

Clinical breast parameters and tumor characteristics were reported before [7]. Briefly, the median tumor size was 10 mm (range 3–19), SBR I-II grade concerned 36 tumors (86%), and 100% of the tumors expressed estrogen receptors. According to our national guidelines, no chemotherapy was indicated for these T1N0 hormone-relevant tumors and adjuvant hormonal treatment was started within the first month for all patients.

No immediate severe side-effect was observed during the IORT procedure. At discharge, three acute wound complications, one infection, five haematoma, and six moderate local pains. None of them necessitated secondary intervention [7].

**Figure 1:**
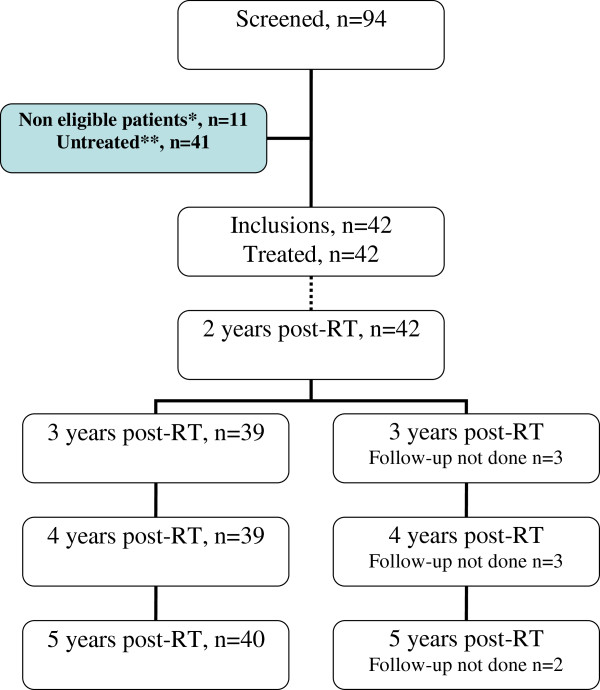
**Consort diagram.** * Eleven 11 patients received IORT but definitive pathology results did not strictly follow the inclusion criteria: positive sentinel nodes were found on the definitive pathology reports for 6 patients; lobular carcinoma was found for 2 patients; bifocality was found in 1 patient; and margins IORT in breast cancer were <2 mm for 2 patients. These two patients with close margins underwent radical mastectomy. After IORT, the 11 patients did not receive any additional external RT and were followed according to the protocol. **The main reasons for nondelivery of IORT (n=41) were: (i) pT/pN restaging during the IORT pathology assessment (n =29), (ii) operative room availability (n = 6), (iii) machine disorder (n = 3), (iv) anesthesia complications (n = 2), (v) informed consent withdrawal (n= 1).

All included patients are still alive with a median follow-up of 72 months (range 66–74, Figure 1). The 5-year-disease free survival is 92.7% (range 79.1−97.6).

Among the 42 included patients, four experienced a local event. Three were defined as ipsilateral breast tumor relapses (IBRT) according to the Royal Marsden criteria [10] i.e. in the same quadrant as the primary tumor, with the same histology and similar or higher grade. These true relapses occurred within the initial tumor bed but relatively delayed after the IORT procedure (25, 54, and 60 months). The two first were 18 and 2 mm ductal unifocal carcinomas. The latter one was considered as a more aggressive relapse identified by five isolated micropapillar tumors but identical to the initial histology.

The fourth local event was a second 3-mm primary ductal carcinoma located in another quadrant and diagnosed 3 months after the surgery. The RADELEC scientific committee considered this event as a second primary tumor omitted during the preoperative staging (staged only by ultrasound).

These four patients underwent standard salvage mastectomy and all restarted a new hormonal treatment for five years more. None received any chemotherapy.

The eleven patients who received IORT but excluded after the definitive pathology results were also followed within the time frame. None of them presented a local recurrence and all are still alive without any disease.

### Long term cosmetic assessment

Post-treatment mammograms showed characteristic pictures of severe cytosteatonecrosis in 30 patients (71%) corresponding to a palpable mass within the IORT area for 17 patients (40%). These structural changes in the tumor bed complicated the evaluation of mammograms within the first two years of follow-up. Two patients required non-routine procedures leading to a biopsy for pathological control. Both of them had outside institution exams.

Ten patients (24 %) reported late grade 1 breast pain. One patient experienced a rib fracture 14 months after the IORT procedure. Globally, 41 among the 42 treated patients (98%) disclaimed a totally satisfaction of IORT and would recommend the procedure.

**Figure 2:**
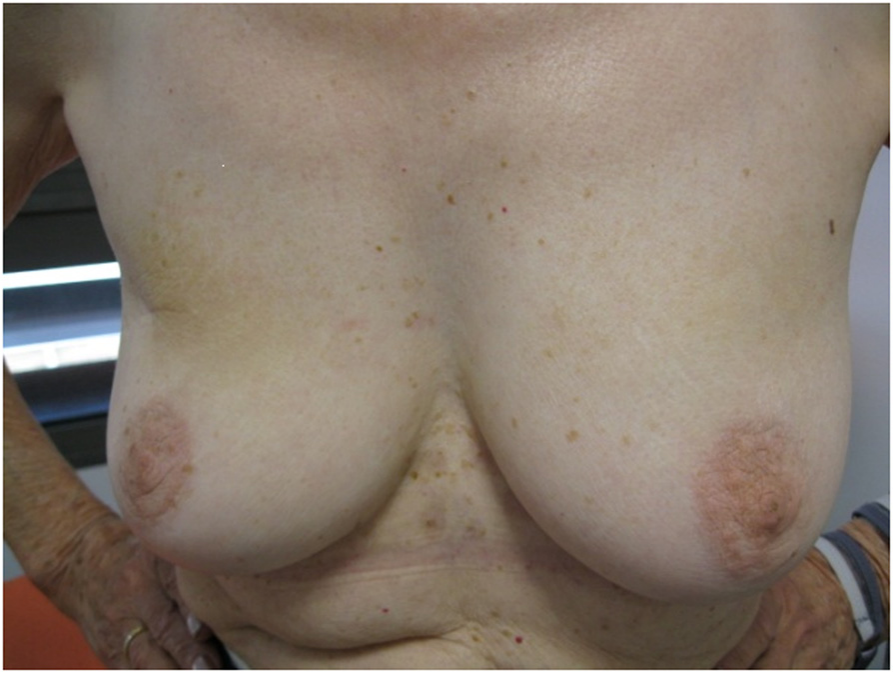
Photographies of patient #1 with 60 months of follow-up.

**Figure 3:**
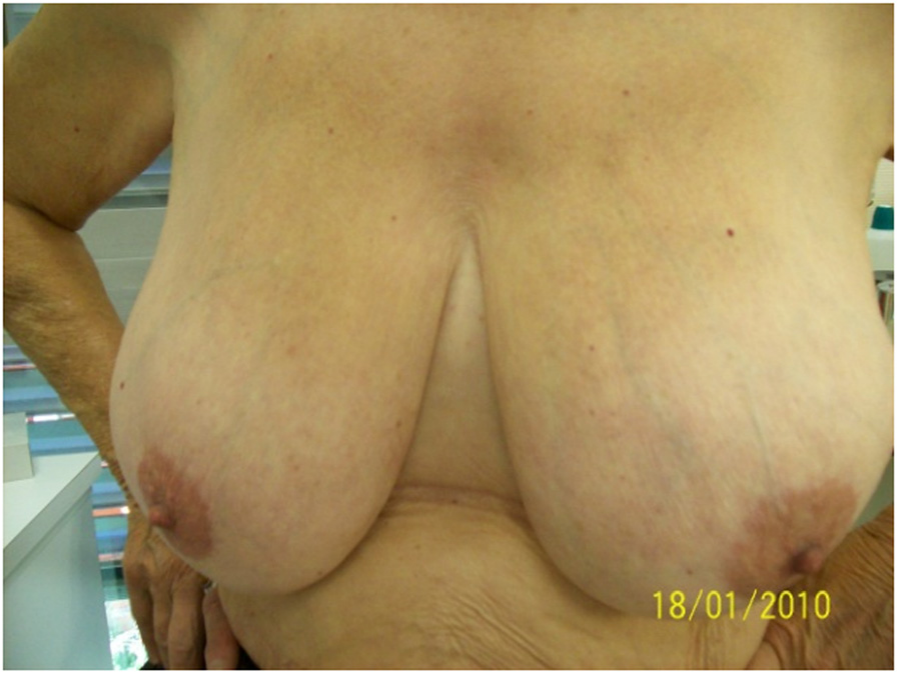
Photographies of patient #2 with 60 months of follow-up.

**Figure 4:**
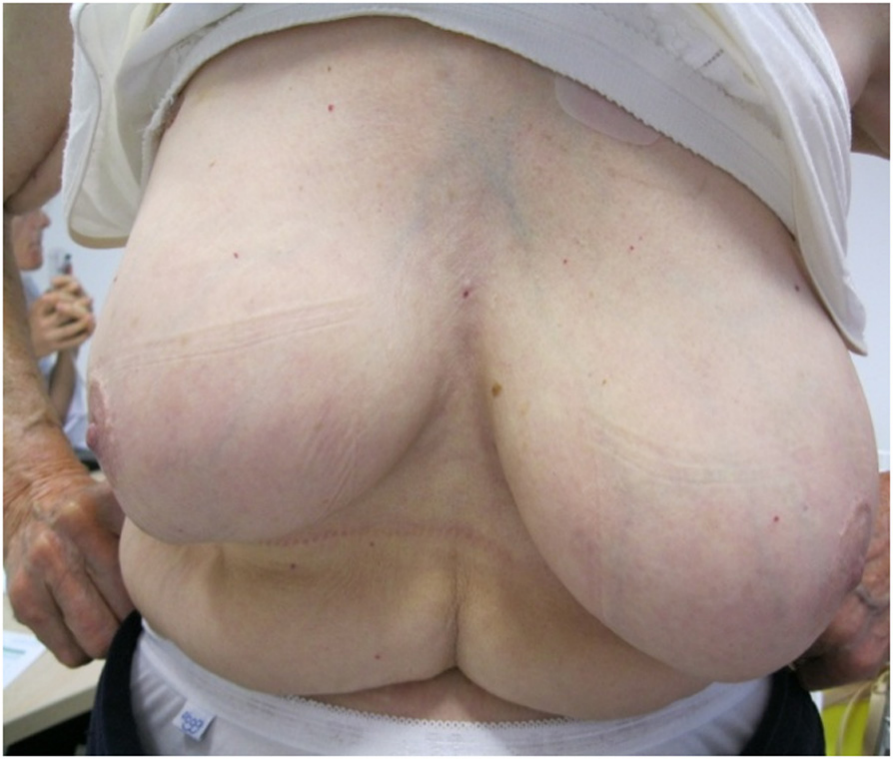
Photographies of patient #3 with 60 months of follow-up.

**Figure 5:**
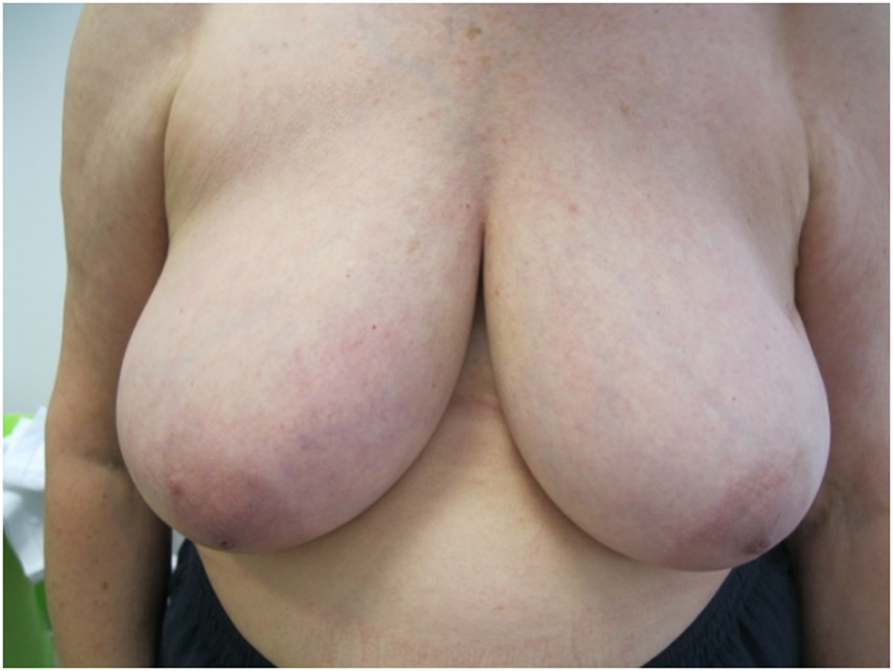
Photographies of patient #4 with 60 months of follow-up.

The 5 year-cosmetic evaluation, including clinical examination and systematic photographs reviewed by 2 physicians showed good to excellent results and are presented for 4 patients in Figures 2, 3, 4 and 5. These photographs showed excellent results whatever the size of the breast (Figure 2, 3, 5). For inferior tumors, scar retraction seems to be higher (Figure 4) but could not be directly attributed to the IORT procedure. The 1, 2, 3, 4, and 5-year cosmetic results were auto-evaluated by patients and by physicians with 97.5% compliance. The results were good and are detailed in Tables 1 and 2. Overall, a poor agreement was observed between patients and physicians about breast size, nipple position, and scar appearance at all evaluation time. In addition, a very poor agreement was observed between patients and physicians about telangiectasia and global cosmetic result whatever the time of evaluation. Regarding breast shape, we observed a poor agreement, a disagreement, and a very poor agreement at 24, 48, and 60 months, respectively. A moderate agreement was observed about nipple shape at 24 months (Table 3). After pooling variables items in two categories (no/small difference vs. middle/high difference), the agreement Kappa coefficient is improved for breast size at 24 and 60 months (0.46 and 0.60, respectively). For breast shape, we observed a strong agreement at 24 and 60 months (0.63 for both) and a perfect agreement for nipple position and nipple shape at 60 months.

**Table 1:** Late cosmetic results from patient evaluation

	**24 m (n=39)**		**48 m (n=39)**		**60 m (n=40)**	
	**N**	**%**	**N**	**%**	**N**	**%**
	**32**	**82.1**	**28**	**71.8**	**28**	**70.0**
**Breast size**						
No difference	20	62.5	20	71.4	20	71.4
Small difference	10	31.3	5	17.9	4	14.3
Middle difference	1	3.1	2	7.1	3	10.7
High difference	1	3.1	1	3.6	1	3.6
**Breast shape**						
No difference	17	53.1	17	60.7	15	53.6
Small difference	13	40.6	8	28.6	10	35.7
Middle difference	1	3.1	2	7.1	2	7.1
High difference	1	3.1	1	3.6	1	3.6
**Nipple position**						
No difference	24	75.0	27	96.4	26	92.9
Small difference	5	15.6	1	3.6	1	3.6
Middle difference	1	3.1	0		0	
High difference	1	3.1	0		1	3.6
Not evaluable	1	3.1	0		0	
**Nipple shape**						
No difference	27	84.4	27	96.4	26	92.9
Small difference	4	12.5	0		1	3.6
Middle difference	1	3.1	1	3.6	0	
High difference	0		0		1	3.6
**Skin color**						
No difference	28	87.5	25	92.6	28	100.0
Small difference	3	9.4	2	7.4	0	
Not evaluable	1	3.1	0		0	
**Scar appearance**						
Soft	16	50.0	14	50.0	14	50.0
Visible, does not affect the result	10	31.3	10	35.7	9	32.1
Visible, slightly affects the result	5	15.6	4	14.3	4	14.3
Visible, significantly affects the result	1	3.1	0		1	3.6
**Telangiectasia**						
Not visible	30	96.8	26	96.3	27	100.0
Rare : <1 / cm^2^	1	3.2	1	3.7	0	
**Global Cosmetic Result**						
Excellent	12	38.7	12	42.9	6	21.4
Good	15	48.4	10	35.7	18	64.3
Fair	3	9.7	5	17.9	2	7.1
Bad	1	3.2	1	3.6	1	3.6
Not evaluable	0		0		1	3.6

*m* months, *N* number.

**Table 2:** Late cosmetic results from physician evaluation

	**24 m (n=39)**		**48 m (n=39)**		**60 m (n=40)**	
	**N**	**%**	**N**	**%**	**N**	**%**
	**32**	**82.1**	**27**	**69.2**	**29**	**72.5**
**Breast size**						
No difference	22	68.8	13	48.2	12	41.4
Small difference	8	25.0	11	40.7	12	41.4
Middle difference	1	3.1	3	11.1	4	13.8
High difference	1	3.1	0		1	3.4
**Breast shape**						
No difference	19	59.4	12	44.4	9	31.0
Small difference	9	28.1	10	37.0	17	58.6
Middle difference	3	9.4	4	14.8	2	6.9
High difference	1	3.1	1	3.7	1	3.5
**Nipple position**						
No difference	24	77.4	18	66.7	17	58.6
Small difference	5	16.1	8	29.6	11	37.9
Middle difference	1	3.2	1	3.7	1	3.4
High difference	1	3.2	0		0	
**Nipple shape**						
No difference	28	87.5	23	85.2	24	82.8
Small difference	3	9.4	4	14.8	4	13.8
Middle difference	0		0		1	3.4
High difference	1	3.1	0		0	
**Skin color**						
No difference	31	96.9	26	96.3	27	93.1
Small difference	1	3.1	1	3.7	2	6.9
**Scar appearance**						
Soft	17	53.1	12	44.4	12	41.4
Visible, does not affect the result	12	37.5	10	37.0	14	48.3
Visible, slightly affects the result	3	9.4	5	18.5	3	10.3
Visible, significantly affects the result	0		0		0	
**Telangiectasia**						
Not visible	32	100.0	27	100.0	28	100.0
**Global cosmetic result**						
Excellent	16	50.0	15	57.7	13	48.2
Good	13	40.6	6	23.1	12	44.4
Fair	2	6.3	5	19.2	1	3.7
Bad	1	3.1	0		1	3.7

*m* months, *N* number.

**Table 3:** Inter-rater agreement (patients and physicians): kappa coefficient

	**24 m (n=39)**	**p**	**48 m (n=39)**	**p**	**60 m (n=39)**	**p**
**Breast size**	0.22	0.063	0.29	0.009	0.19	0.034
**Breast shape**	0.37	0.003	−0.05	0.642	0.12	0.157
**Nipple position**	0.12	0.168	0.14	0.062	0.10	0.111
**Nipple shape**	0.44	0.001	−0.03	0.664	0.04	0.340
**Skin color**	0.37	0.001	−0.04	0.635	−0.02	0.609
**Scar appearance**	0.29	0.016	0.10	0.236	0.29	0.011
**Telangiectasia**	0.00	-	0.00	-	0.00	-
**Global cosmetic result**	0.09	0.23	0.08	0.26	0.04	0.355

*m* months, *n* number.

## Discussion

The updated publication of the collaborative meta-analyses based on individual patient data confirmed that WBRT halves similarly local recurrence rates in the different subgroups of patients and reduces the breast cancer death rate by about a sixth [1].

The need of WBRT was however debated since the publication of the CALGB 9343 trial estimating a low risk of recurrence after conservative surgery in patients older than 70 years presenting HR+ small tumors and only adjuvant tamoxifen as adjuvant treatment [11]. These results were updated at the annual meeting of the American society of clinical oncology (ASCO) in 2010 and showed an increase risk of local recurrence when adjuvant radiotherapy was avoided. At a median follow-up of 10.5 years, 98% of the radiation group and 92% of the tamoxifen-only group were recurrence-free confirming the local risk of avoiding adjuvant irradiation. In addition, the results of the NSABP-21 [12] confirmed that the absolute risk of in-breast recurrences of primary small tumors less than 1 cm is not low enough to spare patients the need for WBRT.

Nevertheless, the standard of 5 to 6 weeks WBRT is no longer the optimized strategy as it appears long and binding in this clinical situation. Indeed, the concept of accelerated whole-breast irradiation recently showed a risk of local recurrences at 10 years of 6.7% among the 612 women assigned to standard irradiation as compared with 6.2% among the 622 women treated in 3 weeks [13]. At the San Antonio Breast Cancer Symposium 2012, the START B trial [14] was updated by Yarnold et al. and confirmed 5.5% and 4.3% of local recurrences at 10 years in the standard and accelerated WBRT, respectively.

Considering that 80% of the local breast recurrences occur within the tumor bed [15], other strategies attempted to reduce the irradiated volume when accelerating the overall treatment time [16]. IORT procedure represents one of the possibilities offering a one-fraction treatment in a limited volume during the primary surgery. Our results confirmed with a long-term follow-up that exclusive partial breast using IORT is feasible for early-breast cancer with an absolute risk of carcinologic events extremely low in much selected patients, namely in the elderly. In that condition, using IORT for partial irradiation with a 21-Gy fraction has the major and unique advantage of a “one-shot” procedure including surgery and radiotherapy at the same time. Extending the duration of surgery, from 20 to 50 minutes, permits to avoid 5 to 6 tiring weeks of external radiotherapy, one of the reasons that encourage patients to decline adjuvant radiotherapy or ask for a mastectomy [17]. This argument is reinforced by the proportion of the patients receiving the recommended adjuvant radiotherapy, less than 75% after seventy and even less than 50% after eighty [18]. IORT may therefore represent an alternative considering the shortness and simplicity of the technique providing that this treatment is done in expert hands.

Our updated results confirm with a long-term follow-up that IORT, as an accelerated partial breast irradiation technique, is suitable for selected patients and could be a better compromise regarding tamoxifen alone in adjuvant setting. However, the local recurrence rates at 6 years, for this very favorable group of patients, seems quite high (9%), especially given the fact that this subgroup of patients with indolent disease will continue to recur in the next 10 and 15 years, for whom rates of 6% at 10 years have been approximated. Therefore, longer follow-up is needed to draw meaningful conclusions.

Alongside our experience in IORT delivered as a boost [4,5], we decided many years ago to extend this concept to a specific very low-risk population (i.e. age ≥ 65 years old, tumor size < 2 cm, non-lobular carcinoma and estrogen receptor positivity) for a unique and exclusive treatment. We excluded BRCA1 or 2 carriers and extensive in situ carcinoma. Since then, the American and European societies for therapeutic radiation oncology (ASTRO and ESTRO, respectively) provided a consensus statement for the use of accelerated partial breast irradiation based on current published evidence and completed by expert opinion [19,20]. The patient selection in the present study is perfectly concordant to these consensuses and reinforces the necessity to respect all the stringent criteria for further studies [21].

The choice of electrons for this partial breast radiotherapy was based on our local experience in this technique [4,5]. We showed that electrons allow an homogenous dose, spare the skin and give time to surgeons for a post-resection reconstruction of the cavity leading to very good cosmetic results. Orecchia et al. presented the 5-year local recurrence rate of patients included in the ELIOT trial (electrons’ technique) at the last World Congress of Brachytherapy in May 2012. The authors observed 3.6% of local recurrence but longer follow-up is warranted to draw definitive conclusions.

Recently, the TARGIT-A trial [22] was published comparing standard WBRT to a single 20-Gy fraction delivered intraoperatively but with a 50-kV system. The 4-year local relapse rates were not inferior in the IORT arm (0.95 and 1.20% in the WBRT and IORT arms, respectively). Mature results were presented at the last San Antonio Breast Cancer Symposium 2012. The investigators continued inclusions after the first publication (from 2232 to 3451 patients) that renders difficult long-term results. The 5-year risks for local recurrence in the conserved breast for TARGIT vs WBRT were 3.3% (95% CI 2.1-5.1) vs 1.3% (95% CI 0.7-2.5). Nevertheless, TARGIT had similar results to WBRT 2.1% (1.1-4.2) vs 1.1% (0.5-2.5) in the pre-pathology subgroup (concomitant surgery and TARGIT) that reinforces the idea that delayed procedure by reopening the wound has to be abandoned. Final data are still pending for publication.

As local recurrences may occur after a long delay, final assessment of kV-IORT will be definitely valid after sufficient follow-up from large international prospective randomized trials.

Frozen section is, for sure, one limiting aspect of any intraoperative procedure, as the definitive pathology report may contradict the biopsy. This technique requires therefore a very close involvement of the pathologist, the surgeon, and the radiation oncologist.

Even if the cosmetic results were evaluated as good, structural changes in the tumor bed after IORT may require a learning curve for the radiologist in order to avoid iterative biopsies [23]. The patient assessments seem extremely important before considering this technique as a standard in daily practice. The use of 50-kV IORT will reduce the risk of late fibrosis and cytosteatonecrosis as the need of dissection is highly less compared to IORT with electrons (71% in the current trial). Indeed, the sphere of Intrabeam fill in the surgical area whereas the tissue surrounding the excision cavity is mo1bilized and approximated by sutures to bring it into the RT planning target volume with electrons.

In contrast, IORT has several advantages and some teams [24] emphasize that using IORT for adjuvant breast radiotherapy may reduce the estimate of second-cancer risk. Compared to classical external WBRT or accelerated partial breast external irradiation, this technique delivers the lowest dose to the controlateral breast, homo-, and controlateral lungs and spine.

Finally, IORT reduces the cost of adjuvant breast radiotherapy and a medico-economic prospective study is still ongoing in France to evaluate the impact of this treatment on healthcare resources and public health.

## Conclusions

In conclusion, our results confirm that IORT given as a sole treatment during breast-conserving surgery is a reliable alternative to conventional postoperative fractionated radiation. This one-stop treatment reduces patient effort and limits the use of health care resources. It could be considered as a standard in a selected population with very-low risk of local recurrence but performed by multidisciplinary expert hands. Patients’ assessments strongly improve long term evaluation of this technique.

## Abbreviations

WBRT: Whole-breast postoperative RT; APBI: Accelerated partial breast irradiation; IORT: Intraoperative radiotherapy; IBRT: Ipsilateral breast tumor relapses; EIC: Extensive intraductal; ER: Estrogen receptor; PR: Progesterone receptor; QI: Quality index; DFS: Disease-free survival; OS: Overall survival.

## Competing interests

All authors declare that they have no competing interests.

## Authors’ contributions

CL, JBD, and MG conceived the study, collected data, and drafted the manuscript. AP, PF, and NA collected data. DA participated in coordination and helped to draft the manuscript. CL, PR, MG, and JBD participated in the design of the study and assisted in data collection.SGB performed the statistical analyses. DA provided mentorship and edited the manuscript. All authors have read and approved the final manuscript.
